# ILNCSIM: improved lncRNA functional similarity calculation model

**DOI:** 10.18632/oncotarget.8296

**Published:** 2016-03-23

**Authors:** Yu-An Huang, Xing Chen, Zhu-Hong You, De-Shuang Huang, Keith C.C. Chan

**Affiliations:** ^1^ College of Computer Science and Software Engineering, Shenzhen University, Shenzhen, 518060, China; ^2^ Academy of Mathematics and Systems Science, Chinese Academy of Sciences, Beijing, 100190, China; ^3^ National Center for Mathematics and Interdisciplinary Sciences, Chinese Academy of Sciences, Beijing, 100190, China; ^4^ School of Computer Science and Technology, China University of Mining and Technology, Xuzhou, 221116, China; ^5^ School of Electronics and Information Engineering, Tongji University, Shanghai, 201804, China; ^6^ Department of Computing, The Hong Kong Polytechnic University, Hong Kong, China

**Keywords:** lncRNAs, functional similarity, disease, cancer, directed acyclic graph

## Abstract

Increasing observations have indicated that lncRNAs play a significant role in various critical biological processes and the development and progression of various human diseases. Constructing lncRNA functional similarity networks could benefit the development of computational models for inferring lncRNA functions and identifying lncRNA-disease associations. However, little effort has been devoted to quantifying lncRNA functional similarity. In this study, we developed an Improved LNCRNA functional SIMilarity calculation model (ILNCSIM) based on the assumption that lncRNAs with similar biological functions tend to be involved in similar diseases. The main improvement comes from the combination of the concept of information content and the hierarchical structure of disease directed acyclic graphs for disease similarity calculation. ILNCSIM was combined with the previously proposed model of Laplacian Regularized Least Squares for lncRNA-Disease Association to further evaluate its performance. As a result, new model obtained reliable performance in the leave-one-out cross validation (AUCs of 0.9316 and 0.9074 based on MNDR and Lnc2cancer databases, respectively), and 5-fold cross validation (AUCs of 0.9221 and 0.9033 for MNDR and Lnc2cancer databases), which significantly improved the prediction performance of previous models. It is anticipated that ILNCSIM could serve as an effective lncRNA function prediction model for future biomedical researches.

## INTRODUCTION

Advances in genome sequencing projects suggest that less than 2% of the human genome encodes protein sequences and the proportion of protein-coding sequence is inversely proportional to the organism complexity. More than 98% of human genome yields a great number of non-coding RNAs (ncRNAs). Specially, long non-coding RNAs (lncRNAs) are heterogeneous ncRNAs with the length of more than 200 nucleotides, which could be divided into five subgroups (i.e. sense, antisense, bidirectional, intronic, and intergenic) according to their relative positions to the coding genes [1–3]. The central dogma of molecular biology assumes that the genetic information is stored in the protein-coding genes. Therefore, lncRNAs were previously considered to be “transcriptional noise” due to their characters of low expression level, high tissue specificity pattern, and low conservation across species [4–6]. However, mounting evidences have indicated that lncRNAs could function as modulators of gene expression network, which challenges the traditional viewpoint on their roles. Specifically, lncRNAs play significant roles in modulating gene expression at the epigenetic, transcriptional, and post-transcriptional levels, getting involved in different biological processes including chromatin modification, cell differentiation and proliferation, RNA progressing, and cellular apoptosis [2, 7–14]. For example, HOTAIR was shown to control the pattern of histone modifications and regulate gene expression by binding to histone modifiers, PRC2 and the LSD1 complex [15]. XIST, a spliced and polyadenylated lncRNA, was shown to bind and recruit PRC2 to initiate X chromosome inactivation [16].

Although the mechanisms of complex diseases are still unclear, experimental observations provide some clues that lncRNAs could carry out functions by gene transcription, chromatin remodeling, interacting with proteins to affect protein activity and localization, or serving as a structural component, which would further accelerate or suppress the development of diseases [17]. Accumulating evidence shows that the dysfunction of plenty of lncRNAs are associated with the development and progression of a wide range of diseases, including cardiovascular disease [18], diabetes [19, 20] and different types of cancers [18, 21–25]. For example, the decreased expression of lncRNA WT1-AS is shown to promote cell proliferation and invasion in gastric cancer [26]. Except for WT1-AS, XIST has been proven to be associated with human glioblastoma stem cells. Knockdown of XIST exerted tumor-suppressive functions by up-regulating miR-512 [27]. HOTAIR is another popularly investigated lncRNA and it has been considered as a potential diagnostic biomarker in various types of human cancers [21, 28, 29].

Due to the rapid development in experimental technology and computational study for lncRNA discovery, there have been thousands of lncRNAs discovered in various eukaryotic organisms ranging from nematodes to humans since H19 and XIST were first identified in the early 1990s [4, 8, 30–32]. Although many lncRNA-related biological datasets have been generated and stored in some publicly available databases, such as NRED [33], NONCODE [34] and lncRNAdb [11], only relatively few lncRNA-disease associations have been collected. Recently, new lncRNA-disease associations have been continually reported from experimental studies. However, considering biological experiments are expensive and time-consuming, it is unrealistic to detect novel lncRNA-disease associations on a large scale based on experimental studies. Based on the assumption that lncRNAs with similar biological functions tend to be involved in similar diseases, some computational models have been proposed to predict novel disease-related lncRNAs, which have drawn increasing attentions [12, 35–39]. By appropriately integrating various types of biological datasets, computational models could provide association probabilities of each lncRNA-disease pairs in a short time and select the most probable association as the candidate for further experiment validation, thus decreasing the time and cost of experimental approaches. Developing computational models not only boosts the understanding of disease mechanism at lncRNA level, but also helps to identify new biomarkers for drug discovery, disease diagnosis, treatment, prognosis, and prevention.

Although the functional impact of several lncRNAs has been confirmed by previous studies, these are just the tip of the iceberg due to the extreme complexity of lncRNA function mechanism. The difficulty of predicting lncRNA functional similarity lies in the function diversity, expression specificity, and current limited understanding of lncRNAs [40]. Some computational models have been proposed to calculate lncRNA functional similarity on a large scale and they can be divided into the following three categories. The first category is based on the lncRNA expression profiles. For example, Chen et al. [36] presented the first lncRNA-disease association prediction model of Laplacian Regularized Least Squares for lncRNA-Disease Association (LRLSLDA). In this study, they defined lncRNA expression similarity as the Spearman correlation coefficient between the expression profiles of each lncRNA pair and then combined it with lncRNA Gaussian interaction profile kernel similarity to obtain integrated lncRNA functional similarity score. Ganegoda et al. [41] reported another method to calculate lncRNA tissue specific similarity by combining Pearson correlation coefficient (PCC) values and expression details of 22 different types of tissues. Methods of the second category integrate other types of biological information, such as epigenetic and transcriptional profiles of lncRNA and lncRNA crosstalk networks. For example, Li et al [42] presented a method to measure the functional similarity between lncRNAs by combining chromatin states data and gene expression patterns. Liu et al. [43] proposed a corpus-based calculation model for the lncRNA functional similarity by considering common target genes. This model is based on the assumption that the functional similarity of lncRNAs is related to the number of their common target genes. To measure lncRNA similarity, Zhou et al. [44] constructed lncRNA-lncRNA crosstalk profiles based on microRNA (miRNA)-associated lncRNA crosstalk networks. The third category is mainly based on the assumption that lncRNAs with similar function tend to interact with similar diseases/miRNAs. Since the lncRNA-disease associations are continually identified with new clinical discoveries, this kind of computational method can make full use of known lncRNA-disease associations. For example, Sun et al. [45] proposed a computational model for calculating lncRNA functional similarity based on an R package named DOsim, which measured semantic similarity between diseases in an ontology sense. For the same purpose of calculating lncRNA functional similarity, Chen et al. [37] developed the model of LFSCM which implemented lncRNA functional similarity calculation based on the integration of known miRNA-disease associations and lncRNA-miRNA interactions. Chen et al. [46] further proposed a novel calculation model called LNCSIM for lncRNA function similarity, which measures the similarity between two lncRNA-associated disease groups to quantify the functional similarity of each lncRNA pair by considering the number of common ancestors of two query disease terms. However, it fails to retain the hierarchical information of Directed Acyclic Graphs (DAGs) of diseases. Besides, even though LNCSIM introduced the concept of information content to retain the specificity of disease terms, simply using the information content values of common ancestors to compute disease similarity could easily suffer from the information bias in DAGs. As another important biological molecule for disease mechanism, calculating functional similarity among miRNAs has become a hot research field. For example, Chen et al. have proposed a calculation model for miRNA functional similarity by adopting a global network similarity measure [47]. Wang et al. have also proposed another computational model which infers miRNA functional similarity by measuring the similarity of their associated disease DAGs [48].

**Figure 1:**
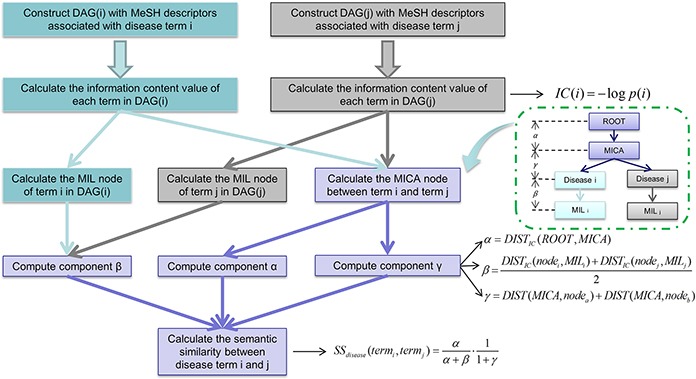
Flowchart of disease semantic similarity function in ILNCSIM based on disease DAGs

**Figure 2:**
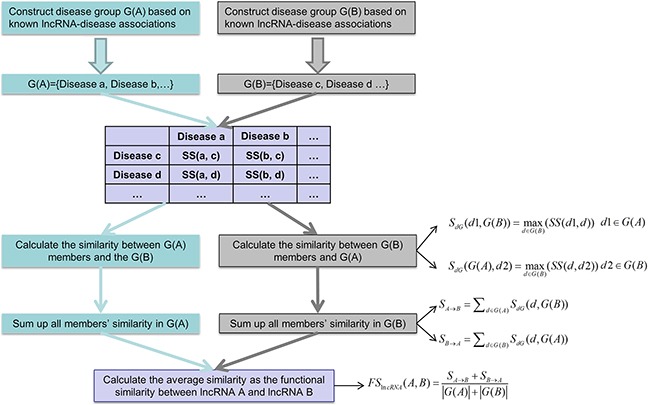
Flowchart of lncRNA functional similarity calculation model based on disease semantic similarity

In this study, we developed Improved LNCRNA functional SIMilarity calculation model (ILNCSIM) based on the assumption that diseases with high similarity tend to be associated with functionally similar lncRNAs and vice versa [36, 49]. ILNCSIM integrates known lncRNA-disease associations and disease DAGs and calculates diseases similarity by an edge-based calculation model. ILNCSIM consists of the following two steps. In the first step, ILNCSIM computes the most informative common ancestors (MICAs) of disease pairs and then calculate their semantic similarities based on the DAG which depicts disease relationship (see Figure 1). Secondly, ILNCSIM further computes the functional similarity of two lncRNAs based on the semantic similarity of disease groups associated with these two lncRNAs (see Figure 2). To further evaluate the performance of ILNCSIM, the calculated lncRNA functional similarity was used to predict the lncRNA-disease associations by combing ILNCSIM with the model of LRLSLDA which was presented in the previous work [36]. The performance of the new model could reflect the effectiveness of ILNCSIM. By adopting the global leave-one-out cross validation (LOOCV) based on manually curated diverse ncRNA-disease repository (MNDR) [50] and Lnc2cancer [51] database, ILNCSIM yielded reliable performance with AUCs of 0.9316 and 0.9074, respectively, outperforming three previously proposed models. For further performance evaluation, we also used 5-fold cross validation, where ILNCSIM yielded average AUCs of 0.9221 and 0.9033 based on MNDR and Lnc2Cancer databases, respectively, higher than the performances of previous models. Besides, 19 lncRNAs in top 20 prediction lists of lung cancer, colon cancer and prostate cancer-related lncRNAs were verified by relevant databases and recent experimental literatures. Based on these reliable results, it is anticipated that ILNCSIM is feasible and effective to quantify the lncRNA functional similarity and has potential value for lncRNA-disease association prediction when ILNCSIM is combined with known similarity-based computational models.

## RESULTS

### Model design

ILNCSIM was developed to quantify lncRNA functional similarity by combining known disease DAGs and known lncRNA-disease associations. In the framework of ILNCSIM, semantic similarity of disease terms and lncRNA functional similarity were calculated in turn. Based on calculated lncRNA functional similarity, similarity-based computational models, such as LRLSLDA and random walk, could be applied to lncRNA-disease association prediction. The reliable performance of ILNCSIM could be largely attributed to the following factors. First, although an alternative approach to estimate the specificity of disease terms has been proposed in LNCSIM by computing information content, this semantic similarity method which uses corpus-based metric has some inherent limitations. Since the known knowledge of a disease term depends on previous studies and the amounts of information of different disease terms are unbalanced, LNCSIM [46] could easily suffer from the bias towards some deeply studied disease terms by only considering information content of disease terms. Besides, hierarchical structure information of DAGs fails to be retained by simply using corpus-based metric. To solve this problem, we developed an edge-based computational model by computing MICAs for retaining the ancestral information of two disease terms by combining the most recent common ancestor (MRCA) and the concept of information content. By computing the distance from MICA to the root node in DAGs, ILNCSIM could depict the hierarchical information of ancestor nodes to a large extent. In addition, the information of distances between disease pairs and the recent common ancestor has been ignored in the model of LNCSIM. The proposed ILNCSIM model retains this middle hierarchical structure by calculating IC-based distances between two disease terms and their MICA. Finally, unlike LNCSIM which only considers the common ancestors, ILNCSIM measures how general two disease terms are in disease DAGs. Specifically, the generality of a disease term is defined as the distance between the term and the most informative leaf terms descending from it.

### Performance evaluation

LncRNA functional similarity scores calculated by ILNCSIM based on MNDR and Lnc2Cancer dataset were listed in Supplementary Table S1 and S2, respectively. The performance of ILNCSIM was evaluated by combining ILNCSIM with LRLSLDA and validating the effectiveness of new model to predict potential lncRNA-disease associations. In the original version of LRLSLDA, lncRNA similarity is generated by combining Guassian interaction profile kernel similarity and lncRNA expression similarity. Here, we integrated new disease similarity generated by ILNCSIM and disease Gaussian interaction profile kernel similarity into the integrated similarity by a simple average operation. A new integrated lncRNA similarity was further calculated based on the lncRNA functional similarity generated from ILNCSIM, lncRNA Gaussian interaction profile, and lncRNA expression similarity. Therefore, we obtained the integrated model named as LRLSLDA-ILNCSIM, which was constructed by two parts, that is, ILNCSIM model yielding disease semantic similarity and lncRNA functional similarity and LRLSLDA model predicting novel lncRNA-disease associations.

Global LOOCV and 5-fold cross validation were implemented based on known detected lncRNA-disease associations in the MNDR and Lnc2Cancer database to evaluate the prediction performance of LRLSLDA-ILNCSIM. By using the global LOOCV method, each known disease-lncRNA association was left out in turn as test sample and all diseases were investigated simultaneously. The test samples whose ranks exceed the given threshold were considered as successful predictions while test samples with ranks lower than threshold were considered to be unsuccessfully predicted. Corresponding true positive rates (TPR, sensitivity) and false positive rates (FPR, 1-specificity) can be obtained by setting different thresholds. Here, sensitivity denotes the percentage of samples whose ranks higher than the threshold and specificity denotes the percentage of samples with lower ranks than the threshold. To plot TPR versus FPR at different thresholds, Receiver-operating characteristics (ROC) curve was drawn for further evaluation. The value of area under ROC curve (AUC) was calculated to quantify the prediction performance of LRLSLDA-ILNCSIM. AUC=0.5 means purely random performance and a higher AUC value means better prediction performance.

**Figure 3:**
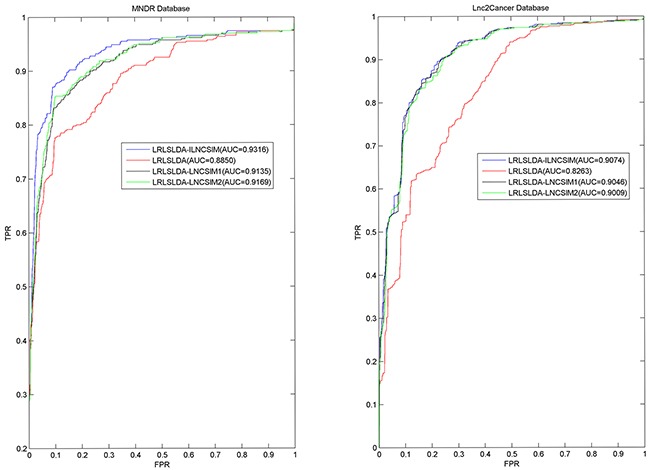
Performance comparisons between ILNCSIM and three the-state-of-art disease-lncRNA association prediction models (LRLSLDA, LRLSLDA-LNCSIM1, and LRLSLDA-LNCSIM2) in terms of ROC curve and AUC based on global LOOCV As a result, ILNCSIM achieved AUCs of 0.9316 and 0.9074 based on the MNDR and Lnc2Cancer databases, which significantly improved all the previous classical models and effectively demonstrated its reliable predictive ability.

LRLSLDA-ILNCSIM was compared with the following three the-state-of-art computational methods in the framework of global LOOCV: LRLSLDA [36], LRLSLDA-LNCSIM1 [46] and LRLSLDA-LNCSIM2 [46] (see Figure 3). As a result, ILNCSIM achieved a significantly improved result with higher AUC than three other existing models. LRLSLDA-ILNCSIM, LRLSLDA, LRLSLDA-LNCSIM1, and LRLSLDA-LNCSIM2 achieved AUCs of 0.9316, 0.8850, 0.9135 and 0.9169 based on the MNDR dataset, and yielded AUCs of 0.9074, 0.8263, 0.9046 and 0.9009 based on the Lnc2Cancer dataset, respectively (see Figure 3).

Furthermore, 5-fold cross validation was used as another validation method for performance evaluation. By using 5-fold cross validation, all known associations were first randomly divided into 5 groups, four of which were used for training and the rest one was used as test samples. To further minimize the influence caused by random division, 5-fold cross validation was repeated 100 times and the mean and standard deviation of AUCs were calculated for fair evaluation. As a result, the best performance based on the MNDR dataset was yielded by LRLSLDA-ILNCSIM with AUC value of 0.9221+/−0.0051. LRLSLDA, LRLSLDA-LNCSIM1 and LRLSLDA-LNCSIM2 yielded relatively poor results with mean AUCs of 0.8687, 0.9012 and 0.9050. For the Lnc2Cancer dataset, LRLSLDA-ILNCSIM, LRLSLDA, LRLSLDA-LNCSIM1 and LRLSLDA-LNCSIM2 yielded average AUCs of 0.9033, 0.8185, 0.9005 and 0.8968, respectively. In conclusion, LRLSLDA-ILNCSIM has proved to achieve performance improvement over existing computational models in the validation framework of global LOOCV and 5-fold cross validation.

### Case studies

Here LRLSLDA-ILNCSIM was applied to three kinds of important cancers based on known lncRNA-disease associations in the MNDR database in order to further validate the effectiveness of ILNCSIM. Prediction results with top 20 ranks were further verified based on two other existing databases, LncRNADisease and Lnc2cancer, and recently published experimental literatures (See Table 1). This evaluation method has been adopted by almost all of the prediction models reviewed in the Introduction section.

**Table 1:** Prediction results of lncRNA associated with colon cancer, lung cancer and prostate cancer in top 20 ranking lists

Disease	lncRNA	Evidence(PMID/Database)	Rank
Colon cancer	H19	Lnc2cancer	1
Colon cancer	UCA1	Lnc2cancer	3
Colon cancer	HOTAIR	LncRNADisease	13
Colon cancer	XIST	Lnc2cancer	14
Colon cancer	MEG3	Lnc2cancer	16
Colon cancer	HULC	Lnc2cancer	19
Lung cancer	BC200	Lnc2cancer	1
Lung cancer	UCA1	26380024	3
Lung cancer	HOTAIR	Lnc2cancer	4
Lung cancer	XIST	Lnc2cancer	8
Lung cancer	GAS5	Lnc2cancer	10
Lung cancer	MEG3	Lnc2cancer	17
Lung cancer	LSINCT5	Lnc2cancer	20
Prostate cancer	H19	LncRNADisease	1
Prostate cancer	CBR3-AS1	LncRNADisease	2
Prostate cancer	UCA1	Lnc2cancer	3
Prostate cancer	KCNQ1OT1	Lnc2cancer	13
Prostate cancer	LINCRNA-P21	Lnc2cancer	14
Prostate cancer	MEG3	LncRNADisease	15

As the third most common cancer worldwide and the most common human malignancies in western countries, the prevalence rate of colon cancer has increased rapidly in recent years [52]. With the development of multidisciplinary research in epidemiology and molecular biology, the understanding of colon cancer etiology has gained a great progress [53]. LRLSLDA-ILNCSIM was applied to predict potential lncRNAs related with colon cancers. As a result, six lncRNAs which have been verified by Lnc2cancer and LncRNADisease databases were predicted as the most potential candidates with top 20 ranks.

Lung cancer is one of the markedly leading causes of death worldwide with about 1.8 million new cases every year. Despite of the development of adjuvant chemotherapy regimens, targeted biologic agents, and understanding on pathophysiological mechanisms, the 5-year survival rate of lung cancer is still dismal [54–56]. With the development of lncRNA-related researches, lncRNAs have been considered as diagnostic and therapeutic targets of lung cancer for therapy studies. LRLSLDA-ILNCSIM was implemented to predict potential lung cancer-related lncRNAs. As a result, seven of top 20 predictions were verified. Specifically, six predicted lncRNAs were proved to be related with lung cancer according to Lnc2cancer database and LncRNADisease database. The association between UCA1 and lung cancer has been confirmed by recent experimental observation that UCA1 provided the highly diagnostic performance for detection of non-small cell lung cancer [57].

It is reported that prostate cancer has become the second most common cancer in men. There were more than one million prostate cancer patients diagnosed and more than 300,000 deaths worldwide in 2012 [58]. Although the pathogenesisof prostate cancer is still unclear, biological experiments prove that the development of prostate cancer is associated with the deregulations of some lncRNAs [53, 59, 60]. We used LRLSLDA-ILNCSIM to predict potential lncRNAs associated with prostate cancer. Among the prediction results, six associations were verified by checking existing databases. Specifically, half of them (H19, CBR3-AS1 and MEG3) were recorded in LncRNADisease database, and three other LncRNAs (UCA1, KCNQ1OT1 and LINCRNA-P21) were recorded in Lnc2cancer database.

To further evaluate the prediction performance of LRLSLDA-ILNCSIM, some statistical data were computed. Specifically, we collected all the lncRNA records which are associated with the three cancers in Lnc2Cancer and LncRNADisease databases, and then removed those records that also exist in MNDR dataset and lncRNAs which are not investigated in this study. As a result, the numbers of remaining lncRNAs which are experimentally confirmed to be associated with colon cancer, lung cancer and prostate cancer are 15, 8 and 9, respectively. The prediction ranks which these lncRNAs obtained based on all the four computational models (i.e. LRLSLDA-ILNCSIM, LRLSLDA-LNCSIM1, LRLSLDA-LNCSIM2 and LRLSLDA) are listed in Table 2, 3 and 4, respectively. We statistically computed the percentage of lncRNAs with top-20 ranks and the average rank of lncRNAs for these three case studies. As a result, the model of LRLSLDA-ILNCSIM yielded the highest percentages of lncRNAs with top-20 ranks, compared with three other models (colon cancer: 40%; lung cancer: 87.5%; prostate cancer: 66.67%). In addition, highest average ranks of lncRNAs were achieved by adopting the LRLSLDA-ILNCSIM model (colon cancer: 40.73; lung cancer: 12.50; prostate cancer: 34.67). This performance comparison indicates that ILNCSIM significantly outperforms all the three previously proposed models.

**Table 2:** Performance comparison between LRLSLDA-ILNCSIM and three other previously proposed models based on the rankings of newly discovered lncRNAs associated with colon cancer, which were recorded in Lnc2Cancer and LncRNADisease databases

LncRNA	LRLSLDA-ILNCSIM	LRLSLDA-LNCSIM1	LRLSLDA-LNCSIM2	LRLSLDA
BACE1AS	192	181	192	38
GAS5	23	54	57	35
H19	1	1	1	2
HOTAIR	13	12	9	6
HULC	19	37	34	31
KCNQ1OT1	24	21	13	93
lincRNA-p21	36	183	73	94
LSINCT5	39	71	78	195
MEG3	16	16	23	10
PRNCR1	67	50	49	83
PVT1	68	190	94	84
uc.338	37	59	52	57
UCA1	3	3	3	4
XIST	14	33	35	33
ZFAS1	59	73	93	88
Percentage in the top 20	40%	26.67%	26.67%	26.67%
Average rank	40.73	65.60	53.73	56.87

**Table 3:** Performance comparison between LRLSLDA-ILNCSIM and three other previously proposed models based on the rankings of newly discovered lncRNAs associated with lung cancer, which were recorded in Lnc2Cancer and LncRNADisease databases

LncRNA	LRLSLDA-ILNCSIM	LRLSLDA-LNCSIM1	LRLSLDA-LNCSIM2	LRLSLDA
BC200	1	1	1	192
GAS5	10	13	21	21
HOTAIR	4	3	4	190
LSINCT5	20	26	39	18
MEG3	17	4	5	74
PVT1	34	63	65	15
UCA1	3	18	11	1
XIST	11	17	18	17
Percentage in the top 20	87.5%	75%	62.5%	50%
Average rank	12.50	18.13	20.50	66.00

**Table 4:** Performance comparison between LRLSLDA-ILNCSIM and three other previously proposed models based on the rankings of newly discovered lncRNAs associated with prostate cancer, which were recorded in Lnc2Cancer and LncRNADisease databases

LncRNA	LRLSLDA-ILNCSIM	LRLSLDA-LNCSIM1	LRLSLDA-LNCSIM2	LRLSLDA
CBR3-AS1	2	11	13	47
H19	1	8	8	225
HULC	26	221	222	211
IGF2-AS	217	216	214	35
KCNQ1OT1	13	38	28	213
lincRNA-p21	14	9	10	54
MEG3	15	7	3	215
NEAT1	21	40	40	7
UCA1	3	1	1	224
Percentage in the top 20	66.67%	55.56%	55.56%	11.11%
Average rank	34.67	61.22	59.89	136.78

The performance achieved in the validation frameworks of global LOOCV, 5-fold cross validation, and case studies has demonstrated the reliable performance of ILNCSIM. Therefore, we further applied LRLSLDA-ILNCSIM to prioritize all the candidate lncRNA-disease pairs based on all the lncRNA-disease associations recorded in MNDR database as training samples. Prediction results were publicly released for further research and experimental validation. (See Supplementary Table S3).

## DISCUSSION

Measuring lncRNA functional similarity is of great benefit to the lncRNA function prediction as well as the potential lncRNA-disease association inference. In this article, we proposed a novel computational model for calculating lncRNA-lncRNA functional similarity based on known lncRNA-disease associations. The functional similarity of each LncRNA pair is measured by the similarity of their associated disease groups based on the assumption that similar lncRNAs tend to be involved in similar diseases. Different from previously proposed models, our model retains the general hierarchical structure information based on an edge-based method. To further evaluate the effectiveness of ILNCSIM, we utilized computed lncRNA functional similarity to quantify lncRNA-disease association probabilities by combining ILNCSIM with LRLSLDA which was proposed in our previous work. By adopting the evaluation methods of global LOOCV and 5-fold cross validation, ILNCSIM-LRLSLDA demonstrated its reliable performance for predicting lncRNA-disease associations. The lncRNA-disease pairs with high ranks could be regarded as validation candidates for further biological experiment confirmation. Therefore, we publicly released potential lncRNA-disease pair for all the diseases investigated in this study. It is anticipated that more predictions with high ranks would be verified by future experiments and that LRLSLDA-ILNCSIM can serve as a pre-experiment method for selecting potential lncRNA-disease association candidates.

There are some limitations in the computational model of ILNCSIM. Firstly, since the degrees of researches for different diseases are imbalanced, the information amount of different diseases recorded in DAGs is different. Diseases of thoroughly-studied topics would have larger information amount which leads to more ancestors and descendants in DAGs than those of uncharacteristic topics. This information imbalance would inevitably result in inaccuracy of disease-based calculation model for lncRNA functional similarity. Even though ILNCSIM uses an edge-based method to alleviate the influence of information imbalance in diseases' DAGs, the calculation result still suffers from the inaccuracy caused by the lack of unrecorded but real lncRNA-disease associations. For example, information bias can mislead the measurement of how specific a disease is. In the proposed model, the average distance between two given diseases and their most informative leaves was computed as component β to evaluate the specificity of diseases (see Figure 1). However, disease terms of thoroughly-studied topics may have more descendants in DAGs, which would lead to overrated specificity with a larger value of component β. Secondly, the final step in disease-disease similarity calculation (i.e. equation 6) can be further optimized by introducing additional constant terms. Finally, the prediction performance of ILNCSIM could be further improved by integrating other types of lncRNA-related and disease-related data from biological databases, such as lncRNA-related various interactions, lncRNA sequence, and disease phenotype information [61]. However, the proposed framework of ILNCSIM fails to integrate additional data for more accurate results. For example, the relationship between lncRNA-disease associations and cancer hallmarks would be a very important problem to address in future studies [62–64]. In particular, a cancer hallmark network could be constructed at the lncRNA levels to effectively evaluate cancer risks.

## MATERIALS AND METHODS

### LncRNA-disease associations

To validate the effectiveness of ILNCSIM, we downloaded human lncRNA-disease associations from the Mammalian ncRNA-disease repository [50] (MNDR, http://www.rna-society.org/mndr/) in March, 2015. The duplicate associations which are verified by different evidence and depict the same lncRNA-disease pair were discarded. As a result, we obtained 471 high-quality experimentally verified human lncRNA-disease associations, including 127 diseases and 241 lncRNAs. To further validate the effectiveness of ILNCSIM, we download a recently collected lncRNA-disease association database called Lnc2Cancer (http://www.bio-bigdata.net/Lnc2Cancer) [51]. This database contains 1057 manually curated lncRNA-disease associations between 531 lncRNAs and 86 human cancers. Similarly, we got rid of those duplicate records and obtained 842 high-quality human lncRNA-disease associations.

### Disease MeSH descriptors

In this work, we utilized disease MeSH descriptors to construct the relations among lncRNA-related diseases. MeSH descriptors were downloaded from the National Library of Medicine (http://www.nlm.nih.gov/) [65]. There are 16 categories of MeSH descriptors including Category A for anatomic terms, Category B for organisms, Category C for diseases, Category D for drugs and chemicals and so on. Here, we used the information of descriptors of Category C. Based on these descriptors, the disease associations can be easily depicted by DAGs where the nodes represent disease MeSH descriptors and edges denote recorded associations among disease terms.

### Disease semantic similarity

Disease semantic similarity was calculated by a novel edge-based method based on diseases' DAGs constructed by their MeSH descriptors. LncRNA functional similarity was then calculated based on disease semantic similarity.

In general, disease terms of higher specificity have a larger contribution to the semantic measurement. Therefore, retaining the specificity of disease terms contributes to the accuracy of calculation model for lncRNA functional similarity. Since information content can effectively measure how specific disease term is, we combined its concept into the common ancestor nodes and the nearest leaf nodes. In the first step for calculating disease semantic similarity, the information content of all diseases was computed. Information content value was computed by computing the negative log likelihood of each term. Given a disease term *a*, its information content is defined as follow:

IC(a)=−log p(a) (1)

In the second step, the MICA node and most informative leaf (MIL) were computed based on the IC-based distances which could be defined as follow:

$$\text{DIS}T_{\text{IC}}(a,b)=\left|\text{IC}(a)-\text{IC}(b)\right|$$ (2)

where *a* and *b* denotes two different disease terms. MICA node denotes the common ancestor node with the smallest IC-based distance, and MIL node denotes the leaf node with the smallest IC-based distance. By this way, the MICA node and the MIL nodes were computed and considered as the key nodes for retaining the general information of DAGs.

In the third step of disease similarity measurement, three different components (i.e. α β and γ) were computed based on computed nodes of MICA and MILs. Component α measures the specificity of MICA based on the IC-based distance from the root node. Component β measures the generality of two query disease terms based on the IC-based distance from the MIL nodes. Component γ measures the sum of IC-based distances between two terms and their MICA.

The component α, β and γ can be further computed as follows:

$$\alpha =\text{DIS}T_{\text{IC}}(\text{ROOT},\text{MICA})=-\text{log }p(\text{MICA})$$ (3)

$$\beta =\frac{\text{DIS}T_{\text{IC}}(\text{nod}e_{a},\text{MI}L_{a})+\text{DIS}T_{\text{IC}}(\text{nod}e_{b},\text{MI}L_{b})}{2}$$ (4)

$$\gamma =\text{DIST}(\text{MICA},\text{nod}e_{a})+\text{DIST}(\text{MICA},\text{nod}e_{b})$$ (5)

where ROOT denotes the root node in DAGs; MIL_a_ and MIL_b_ represent the most informative leaf node of node *a* and node *b* respectively.

In the final step, the semantic similarity of two disease terms were calculated by combing α, β and γ:

$$\text{SS}(\text{ter}m_{a},\text{ter}m_{b})=\frac{\alpha }{\alpha +\beta }\cdot \frac{1}{1+\gamma }$$ (6)

In this way, IC-based distances help to search the key nodes for more accurate measurement and the overall structure information in DAGs which contributes the semantic similarity measurement can be retained to a large extent. Clearly, *SS*=0 (α=0) indicates that the MICA of term_a_ and term_b_ is the root of DAG, which accords with the assumption that two terms share no commonality in biological functions without common ancestor nodes. On the other hand, *SS*=1 (β=0 and γ=0) indicates that term_a_ and term_b_ are the same leaf term. By calculating the semantic similarity of each disease pairs, disease similarity matrix *SS* can be constructed, where the entity in row *m* column *n* depicts the semantic similarity between disease *m* and disease *n*.

### ILNCSIM

Based on the semantic similarity of diseases, we developed the model of ILNCSIM to calculate the functional similarity among lncRNAs by considering their associated disease groups. Given two disease groups, G(i) and G(j), associated with lncRNA i and lncRNA j, respectively, we computed similarity between G(i) and G(j) as the functional similarity of two given lncRNAs. Here, a group-based measure method was proposed to calculate similarity between two disease groups. The similarity between one of disease terms in G(i), such as d1, and G(j) were defined as follow:

$$S_{\text{dG}}(d1,G(j))=\underset{d\in G(j)}{\text{max}}(\text{SS}(d1,d))$$ (7)

The general similarities of two groups to each other were then computed by a sum operation as follows:

$$S_{i\to j}=\sum_{d\in G(i)}S_{\text{dG}}(d,G(j))$$ (8

$$S_{j\to i}=\sum_{d\in G(j)}S_{\text{dG}}(d,G(i))$$ (9

Finally, the functional similarity between lncRNA *i* and lncRNA *j* was further defined as follow:

$$FS_{\text{ln}c\text{RNA}}(i,j)=\frac{S_{i\to j}+S_{j\to i}}{\left|G(i)\right|+\left|G(j)\right|}$$ (10)

where |G(i)| and |G(j)| denote the numbers of disease in G(i) and G(j), respectively.

### Webserver of ILNCSIM

For convenient application, we built a web server which implements the function of the proposed ILNCSIM model. This web server is freely available at http://219.219.60.246/ILNCSIM/. This web server presents similarity calculation based on ILNCSIM and two lncRNA-disease association databases (i.e. MNDR and Lnc2Cancer). More importantly, this webserver could provide similarity calculation function for new lncRNAs with associated diseases provided by users. When visitors provide a specific lncRNA with its associated diseases, the functional similarity between this query lncRNA and all lncRNAs in two databases would be computed and listed on the webpage.

## SUPPLEMENTARY MATERIALS TABLES








