# Supplementary material for: ILNCSIM: improved lncRNA functional similarity calculation model
Source: Oncotarget. 2016 Mar 23;7(18):25902–14. doi: 10.18632/oncotarget.8296 (PMC5041953; doi:10.18632/oncotarget.8296)
Supplement: Supplementary file 1 [file oncotarget-07-25902-s001.pdf]

SUPPLEMENTARY METERIAL

Supplementary Table S1:LncRNA functional similarity scores calculated by ILNCSIM based on MNDR dataset.

(See Supplementary File S1)

Supplementary Table S2: LncRNA functional similarity scores calculated by ILNCSIM based on Lnc2Cancer dataset.

(See Supplementary File S2)

Supplementary Table S3: We further applied LRLSLDA-ILNCSIM to prioritize all the candidate lncRNA-disease pairs based on all the lncRNA-disease associations recorded in MNDR database as training samples. Prediction results were publicly released for further research and experimental validation.

(See Supplementary File S3)
